# An Objective Comparison of the Quality and Reliability of Information Regarding Autism Spectrum Disorder on YouTube

**DOI:** 10.7759/cureus.60904

**Published:** 2024-05-23

**Authors:** Deeksha Chakrabarty, Nikhil Arora, Iffath Muneer Ahmed, Sruthi Satish

**Affiliations:** 1 General Practice, Kasturba Hospital, Manipal, IND; 2 Pediatrics, Government Medical College Patiala, Patiala, IND; 3 Pediatrics, Guru Gobind Singh Medical College and Hospital, Faridkot, IND; 4 Family Medicine, Shadan Institute of Medical Sciences, Hyderabad, IND; 5 Internal Medicine, Kasturba Medical College, Manipal, Manipal, IND

**Keywords:** reliability score, global quality score, social media, youtube, autism

## Abstract

Background: YouTube serves as a good source of information on autism; however, the reliability and quality of such content remain uncertain. This study aimed to evaluate the reliability and quality of autism-related information presented in YouTube videos using the Global Quality Score (GQS) and Reliability Score.

Methods: A cross-sectional observational study was conducted in November 2023. A total of 48 autism-related videos on YouTube were sourced using keywords such as ‘autism’, ‘autism cause’, ‘autism treatment’, and ‘autism kids’. The authors then viewed the videos and collected data regarding the number of views, likes and comments, uploader type, and type of information disseminated. The authors also used The GQS and modified DISCERN score to assess the quality and reliability of information in the videos. The data was then subjected to statistical analysis using the Kruskal-Wallis test and IBM SPSS Statistics for Windows, Version 22 (Released 2013; IBM Corp., Armonk, New York, United States).

Results: Out of 48 videos, seven videos were excluded, leaving 41 for analysis. The included videos amassed 25,540,635 views, 304,557 likes, and 37,039 comments. The majority of videos were uploaded by hospitals (n=15; 36.59%), followed by news channels (n=12; 29.27%). Most videos described autism symptoms (n=26; 63.41%), with fewer addressing potential etiology (n=16; 39.02%). The median GQS was highest for videos uploaded by healthcare professionals (n=5), contrasting with news channels. The Kruskal-Wallis test revealed significant differences (p=0.02).

Conclusion: These videos collectively garnered substantial viewership, likes, and comments. Most videos described autism symptoms, although fewer addressed potential causes. Notably, videos uploaded by healthcare professionals achieved the highest GQSs, highlighting their significance in disseminating reliable autism information. Healthcare professionals therefore play a crucial role in disseminating reliable autism information via YouTube. Encouraging their involvement in creating informative videos can enhance public understanding of autism.

## Introduction

Autism spectrum disorder (ASD) is a neurobehavioral condition and developmental disability that leads to difficulties in social relationships and communication. Individuals with ASD also exhibit repetitive behaviors, narrow interests, and unusual reactions to sensory inputs. Typically diagnosed in children before the age of five, ASD persists throughout life and is more prevalent in boys than girls. Despite the challenges associated with ASD, appropriate support can enable individuals to fully engage in various aspects of society. Diagnosing ASD is complex due to the absence of a specific test; experienced medical practitioners like psychiatrists and pediatricians rely on the DSM-5 to make diagnoses based on behavioral observations [1].

The rapid growth of multimedia and social media has transformed how information is disseminated and consumed, influencing public perceptions and behaviors. YouTube, a widely used American social media platform for video sharing, plays a significant role in this context. Users leverage YouTube for a variety of purposes, including the dissemination of health-related information [2]. However, the abundance of information that may or may not be false makes it difficult for individuals to find trustworthy sources of information, as observed in the coronavirus disease 2019 (COVID-19) pandemic. The most detrimental effects of spreading health misinformation include the rise in erroneous or confusing interpretations of the existing data and improper use of available resources.

Thus, understanding the quality of information available on YouTube about ASD is crucial, given the platform's extensive reach and influence. Although the precise causes of ASD remain unknown, it is considered a multifactorial disorder influenced by environmental, genetic, and epigenetic factors [3]. Diagnosis relies on expert behavioral observations, and while ASD cannot be cured, it can be managed with early intervention programs focusing on social and communication skills; any medications are typically prescribed for co-occurring conditions such as attention deficit hyperactivity disorder and anxiety, rather than ASD itself [4].

Previous research has highlighted the variability in the quality of information available on YouTube, particularly regarding health-related topics. For ASD, the primary subjects in YouTube videos are often autistic children, teenagers, and adults. Most content focuses on symptoms and signs, with professional sources more frequently addressing diagnosis and available resources. Despite this, all videos, regardless of source, have been found to have poor understandability and actionability scores, although professionally produced videos tend to be better understood [5]. Thus, the present study intends to fill a gap in the literature regarding the evaluation of ASD-related information on a widely accessed social media platform, thereby contributing to improved public understanding and resource utilization.

Aims and objectives

This study aimed to assess the quality and reliability of information about autism on YouTube using the Global Quality Score (GQS) and a modified DISCERN score. Additionally, it sought to identify any associations between the uploader type and the quality and reliability of the content.

## Materials and methods

This study adopted a cross-sectional observational design conducted over a span of four weeks in November 2023. All data was collected from publicly available sources on YouTube and did not involve any confidential human participant data. Thus, the study was deemed exempt from ethical approval.

The investigation was carried out exclusively with YouTube videos using specific keywords such as “autism”, “autism cause”, “autism treatment”, and “autism kids” to ensure relevance to the topic. 

Inclusion criteria included videos containing content related to autism in either English or Hindi language, with durations ranging from one to twenty minutes. Selection of videos was undertaken collectively by the authors, who independently assessed 10 videos each, with repeated entries removed. Data collection occurred over a three-day period, concluding on November 18th, 2023. 

The authors viewed the videos, and information regarding the number of views, likes, and comments at the time of data collection was recorded for each video. Information regarding the uploader was also collected, i.e. the type of uploader (Doctor/Hospital/Health Organization/News Agency/Patient, etc.). The type of content being discussed in the videos was also recorded.

Quality and reliability of the content discussed in the videos were analyzed by the authors using the GQS and the modified DISCERN score [6,7]. The GQS scale, ranging from 1 to 5, was employed to evaluate the relevance and quality of the content. A score of 1 indicated poor quality, while a score of 5 denoted excellent quality. This scale enabled the quantification of the overall quality of information conveyed in autism-related YouTube videos [6].

A modified version of the DISCERN scale was also utilized to evaluate the dependability and excellence of health information. This scale assessed variables such as the precision of data, clarity of explanation, and reliability of references. Each "yes" response received 1 point, while each "no" response received 0 points. A higher score on the modified DISCERN scale indicated a higher level of information quality [7].

The data thus collected was then exported to Microsoft Excel (Microsoft Corporation, Redmond, USA) for further analysis. IBM SPSS Statistics for Windows, Version 22 (Released 2013; IBM Corp., Armonk, New York, United States) was used to perform statistical analysis, with the Kruskal-Wallis test employed to identify any statistically significant associations between the GQS and modified DISCERN scores with uploader type. A p-value <0.05 was considered statistically significant.

## Results

A total of 48 videos on autism were evaluated on YouTube, of which seven videos were excluded, as they did not meet our inclusion criteria, leaving 41 videos to be included in our study. 

The total views of the included videos were 25,540,635; the likes were 304,557 and the total number of comments was 37,039. Table 1 shows the characteristics of YouTube videos analyzed. It was seen that the maximum number of videos were uploaded by hospitals (n=15; 36.59%) followed by news channels (n=12;29.27%) and then doctors (n=5;12.20%).

**Table 1:** Characteristics of YouTube videos analyzing autism.

Characteristics	N (%)
Time since uploaded	
More than a week to last one year (<365 days)	9 (21.95)
More than one year (>365 days)	32 (78.05)
Popularity	
Total no. of views	25,540,635
Total no. of likes	304,557
Total no. of comments	37,039
Type of uploader	
Doctor	5 (12.20)
Hospital	15 (36.59)
Healthcare organization	4 (9.76)
News channel	12 (29.27)
Parent of a patient suffering from disease	4 (9.76)
Patient suffering from disease	1 (2.44)

Table 2 depicts the type of information circulated about the disease in the YouTube videos. The majority of videos described the symptoms of autism (n=26;63.41%), followed by 39.02% (n=16) of them with information about the cause. 

**Table 2:** Information about “autism” in the YouTube videos.

Variables	N (%)
Description of symptoms	26 (63.41)
Information about cause/etiology?	16 (39.02)
Information about investigations/tests	8 (19.51)
Information about prevention/vaccines	1 (2.44)
Information about treatment	15 (36.59)
Information about rehabilitation	4 (9.76)
Information about support groups	1 (2.44)
Information about people/patient’s sharing their own experience	10 (24.39)
Information about parent sharing their experience with their family members	5 (12.20)
The post has a promotional content by pharmaceutical company or by doctors?	2 (4.88)

Table 3 shows a comparison of the GQS and Reliability Score based on the type of uploader. The median GQS was found to be maximum (GQS = 5), for the videos uploaded by doctors and least (GQS = 2) for the news channels. The Reliability Score was found to be highest among doctors, hospitals, patients, and patient relatives/guardians. The results were statistically significant (p=0.02) for the GQS, whereas the results for the reliability score for videos uploaded by various groups were not significant statistically.

**Table 3:** Comparison of the Global Quality Score and modified DISCERN (Reliability) Score based on the type of uploader. Values are mentioned as median (IQ1, IQ3) where IQ stands for the interquartile range. *p-value <0.05 is statistically significant.

Type of Uploader/Scoring Tool	Doctors (n=5)	Hospital (n=15)	Healthcare organization (n=4)	News channel (n=12)	Parent of a patient with autism spectrum disorder (n=4)	Patient with autism spectrum disorder (n=1)	Kruskal-Wallis test
Global Quality Score	5 (3,5)	3 (2,4)	3 (2.5,3.5)	2 (2,3)	4 (4,4)	4 (4,4)	0.02*
Reliability Score	3 (3,4)	3 (2,3)	2.5 (2,3.5)	2 (1.5,3)	3 (3,3)	3 (3,3)	0.27

## Discussion

An infodemic is characterized as an abundance of information that may or may not be false which makes it difficult for individuals to find trustworthy sources of information. This phenomenon was most recently described in the context of the COVID-19 pandemic by the WHO, with the most detrimental effects of spreading health misinformation including the rise in erroneous or confusing interpretations of the existing data and improper use of available resources [8]. The proliferation of inaccurate health information causes delays in the delivery of care and a rise in the use of divisive and hostile speech [9]. This study aimed at objectively analyzing the quality and reliability of content available about autism on the social media platform, YouTube.

The authors analyzed 48 videos with a total number of 25,540,635 views, 304,557 likes, and 37,039 comments. Most videos examined by the authors were uploaded by hospitals (36.59%), consistent with Bellon-Harn et al., who additionally found that the mean number of views on videos posted by professionals was greater than consumer and Internet-based videos [5]. In contrast, Kollia et al. observed only a single video posted by a professional. This variance may be accounted for by the difference in search terms utilized to obtain data [10].

Data analysis revealed that 63.41% of the videos inspected provided a description of the symptoms and 36.59% of the videos provided information relating to the treatment of the disorder. The importance of accurate information regarding early identification of symptoms and available treatment options is highlighted by the fact that early intervention in ASD has been linked to better outcomes [11]. Information about support groups and rehabilitation was only present in one and four of the videos (2.44%) in the sample respectively. This calls attention to the need to supply easily accessible resources that adequately equip individuals with autism and their caregivers to cope with the diagnosis in day-to-day life [12]. Similar findings were illustrated by Cavalcante et al., who indicated that some of the videos they examined failed to provide supplementary sources of information [13].

Furthermore, 10 (24.39%) videos among the sample feature individuals with autism describing their own experience, and five videos (12.20%) present caregivers of patients recounting their experiences with the disorder. The importance of such content was highlighted by Naslund et al., who found in their study that videos providing anecdotal evidence such as these greatly benefit people living with mental illness as they provide a feeling of connectedness and belonging [14]. Furthermore, these videos have an extensive reach and promote the establishment and maintenance of therapeutic alliances with mental health professionals.

The quality and reliability of the sample videos were objectively evaluated using the GQS and the Reliability Score (derived from the DISCERN tool for assessment of written health information) respectively. Suresh et al. and Dobosz et al. conducted comparable analyses regarding anorexia nervosa and body dysmorphic disorder respectively, to evaluate the standard and utility of the information published online [2,15]. The results were found to be statistically significant only about the quality of the videos measured by GQS. Videos issued by doctors were found to be the most reliable with a mean GQS of 5 and the lowest reliability was found to be among videos issued by news channels with a mean GQS of 2. It is also noteworthy that videos uploaded by patients or parents of patients suffering from autism are of comparatively higher quality with a mean GQS of 4. The analysis also identified only two videos displaying promotional content by a company or by a doctor; hence, the caliber of the videos assessed has not been compromised by concerns of monetary gain. Cavalcante et al. comparably reviewed Brazilian-Portuguese content on ASD and analyzed the quality and trustworthiness using GQS and the DISCERN checklist [13]. They classified the videos they evaluated into experiential and informative subtypes and found that most of the informative videos demonstrated high-quality and trustworthy material [13].

Limitations

While the present study offers insights regarding the quality and reliability of information regarding ASD, it is limited by the small sample size and the languages in the videos selected for analysis, namely English and Hindi. Observer bias may affect the qualitative data collected by authors. Lastly, the context in which the videos were uploaded, which tends to affect the content of the videos, was not considered.

## Conclusions

Social media contains a wide array of information regarding ASD, ranging from symptomatology to treatment and rehabilitation options. The easy accessibility of this information is very convenient for patients and their caretakers who can use this wealth of information to their advantage. The videos assessed in the present study collectively garnered substantial viewership, likes, and comments. Most videos described autism symptoms, although fewer addressed potential causes. Notably, videos uploaded by healthcare professionals achieved the highest global quality scores, highlighting their significance in disseminating reliable autism information. Healthcare professionals therefore play a crucial role in disseminating reliable autism information via YouTube. Encouraging their involvement in creating informative videos can enhance public understanding of autism.

The availability of this data also leads to social inclusivity of individuals living with mental illness, particularly when the subject is discussed by peers who have been through similar experiences. However, the accuracy of the available material remains uncertain, and the policing of inaccurate information presents a complicated task. The authors encourage mental health professionals to make videos to provide information about and to reduce public stigma about autism and individuals with the disorder. The importance of verifying the information published using standard metrics of quality and reliability cannot be overstated as the impact of the content created is far-reaching.
